# Location Matters—Can a Smart Golf Club Detect Where the Club Face Hits the Ball?

**DOI:** 10.3390/s23249783

**Published:** 2023-12-12

**Authors:** Bernhard Hollaus, Yannic Heyer, Johannes Steiner, Gerda Strutzenberger

**Affiliations:** 1 Department of Medical, Health & Sports Engineering, MCI, Maximilianstraße 2, 6020 Innsbruck, Austria; yannic.heyer@gmail.com; 2 Johannes Steiner Golf, Robert-Fuchs-Str. 40, 8053 Graz, Austria; golf@johannes-steiner.at; 3 Institute for Sports Medicine Alpine Medicine & Health Tourism (ISAG), UMIT TIROL—Private University for Health Sciences and Health Technology, Eduard-Wallnoefer-Zentrum 1, 6060 Hall in Tirol, Austria; gerda.strutzenberger@umit-tirol.at; 4 MOTUM—Human Performance Center, Steinbockallee 31, 6063 Rum, Austria

**Keywords:** golf, swing analysis, impact analyses, convolutional neural network, machine learning

## Abstract

In golf, the location of the impact, where the clubhead hits the ball, is of imperative nature for a successful ballflight. Direct feedback to the athlete where he/she hits the ball could improve a practice session. Currently, this information can be measured via, e.g., dual laser technology; however, this is a stationary and external method. A mobile measurement method would give athletes the freedom to gain the information of the impact location without the limitation to be stationary. Therefore, the aim of this study was to investigate whether it is possible to detect the impact location via a motion sensor mounted on the shaft of the golf club. To answer the question, an experiment was carried out. Within the experiment data were gathered from one athlete performing 282 golf swings with an 7 iron. The impact location was recorded and labeled during each swing with a Trackman providing the classes for a neural network. Simultaneously, the motion of the golf club was gathered with an IMU from the Noraxon Ultium Motion Series. In the next step, a neural network was designed and trained to estimate the impact location class based on the motion data. Based on the motion data, a classification accuracy of 93.8% could be achieved with a ResNet architecture.

## 1. Introduction

Technology became increasingly present throughout sports in recent decades. The need for more and better information about an athlete’s performance made digitalmeasurement systems ubiquitous. As a result, professional athletes are monitored regularly during training, competitions, and also outside of their sporting activities with the major goal to optimize performance and receive direct feedback. Amateur athletes also desire more and better information on their performance, but usually have limited possibilities to measure and analyze it. One major limitation is whether the measurement devices are mobile or stationary. A professional athlete is more likely to have infrastructure and staff that enables monitoring in contrast to an amateur athlete. Therefore, amateur athletes are more likely to use mobile measurement devices to derive their performance, as the accessibility of them is better in comparison to the stationary ones. Nevertheless, mobile measurement devices are also limited to a group of sports.

Running, cycling, ski touring, tennis, and other sports which have a repetitive motion pattern and usually take place in a large area outside, often utilize smart devices for deriving performance criteria [1,2,3,4]. Based on motion and global position data, performance indicators the stride length can be calculated or estimated [5]. Many devices additionally measure the heart rate, SPO2 and similar variables continuously to make even better estimations on the performance in endurance sports [6]. In general, the use of smart devices became more common in recent years, and along with this development went the development of analysis methods.

In 2020, the digi sporting consortium published a summary of electronic performance tracking systems (EPTS), based on several classes of tracking methods [7]. The major share of the systems were based on one of two major methods for measuring motion. Either they used optical tracking within the EPTS, or the measurements were based on inertial measurement units (IMU). Both approaches deliver motion data for further analysis, usually done by sport-specific algorithms. As an example, companies like Statsports use IMU and global navigation satellite system (GNSS) data to determine the performance in sports like field hockey, rugby, soccer and many other sports [8,9]. Memmert, Park and Jackson utilized optical methods to determine the positions of athletes and their change over time [10,11,12] to derive performance criteria in their studies. Although optical methods are widely spread in elite sports to derive various performance metrics, amateur sports often choose an IMU- and GNSS-based approach packed in some sort of smart device like a smartwatch, fitness tracker or similar. A major reason for that is the acceptance of such devices in various sports because of the easy accessibility of the devices.

In cricket, the team around Fuss used IMUs to create a smart cricket ball, and derive performance metrics from the recorded data [13,14]. McGrath et al. also used a smart IMU-based cricket ball in combination with machine learning to derive bowling speed [15]. In general, there are several manufacturers that released smart balls or smart sensors for clubs, rackets or similar, that are equipped with IMUs. In football, various papers reference the adidas micoach. In 2019, Kryger et al. compared different systems for measuring the velocity of footballs, including the adidas micoach [16]. Also, in the research on measuring impacts to the head during headers in football, Stone used the micoach [17]. Other approaches have the IMU placed in the football boot or somewhere on the lower extremities to derive motion and performance data [18,19]. Other systems in golf also rely on IMU data. An example is the smart golf ball GEN i1 of coachlabs [20]. A major limitation is the restricted usage for putting. All the mentioned papers and products use IMUs to measure performance in terms of motion. Unfortunately, they do not give any information on the impact location on the hitting element (head, leg, hand, club), which is a major performance criterion in golf, especially for a novice athlete.

A sport that has several IMU-based systems to derive the impact location from the gained data are tennis. The known geometry of the racket in combination with the position of the IMU allows a link between motion data and impact location. Most of the mentioned systems in [21] use some IMU in combination with algorithms to determine the impact location. This approach could be transferable to golf (e.g., Jensen provided an IMU-based system in golf in the year 2015 for putting analysis). The system potentially had all relevant sensors to derive the impact location, but their experiment did not focus on it. Other research in tennis, where the interaction between tennis racket and ball is from high importance, used machine learning approaches to classify shots based on IMU data [22]. The research group applied a modified version of the ResNet architecture, introduced by [23] and adapted from the original ResNet architecture from [24].

Current IMU-based systems have been combined with state-of-the-art signal processing methods to extend the possibilities in performance measurement in recent years [25,26]. Sports that do not have a repetitive motion pattern, but rather have an impact nature in combination with sports gear, have especially been researched by various institutions [27,28]. This is also true for golf, when it comes to the measurement of the human motion of the golf club. Performance characteristics for the handling of a golf club could be determined within the experiments of Kim et al. or Huang et al. [29,30]. Biomechanical analysis has been done by Cole et al., Chu et al. and Zhou et al. over the last decade, delivering results for the optimal swing motion [31,32,33]. Nevertheless, the focus within the IMU-related research was not towards the interaction of the golf club and the ball.

The major aspect of this paper is to gain further information about the location of the interaction of golf ball and golf club, as the variance of it throughout several golf swings can be used as a performance criterion [34,35]. Betzler et al., point out the significance of variability in impact location for determining both launch angle and total distance variability [36]. Using numerical analysis, Iwatsubo et al., identified a sweet spot or sweet area, where the release velocity of the ball is maximal compared to other impact areas on the club surface [37], highlighting the importance of the impact location for the total distance of a drive.

Optical systems that also consider radar technology are state-of-the-art in performance analysis in amateur golf. Devices like the TrackMan 4 from the company TrackMan already deliver information on the impact location [38], with the major drawback of being a stationary solution. Mobile solutions, such as the Mobile Launch Monitor of Rapsodo also use the optical approach in combination with radar sensors. According to the technical specifications, the device does not further investigate the impact location. Also, mobile optical- and radar-based measurement systems are prone to limited accuracy due to inconsistent conditions, such as illumination, humidity and other factors.

Due to the given reasons, the development of a mobile IMU and machine learning (ML)-based measurement system, that delivers performance information of the impact location could push the current state of the art. Therefore, the authors want to provide a proof of concept that it is possible to determine the impact location of a golf ball on a golf club, based on IMU data from the golf club.

## 2. Materials and Methods

Within this section, the phases of the project are covered in chronological order. In the first stage, the experiment had to be designed and carried out so relevant data could be recorded. In the second stage, the data needed further processing and augmentation to enable the development of an algorithm that estimates the point of interaction based on the collected data.

### 2.1. Experimental Design

**Figure 1:**
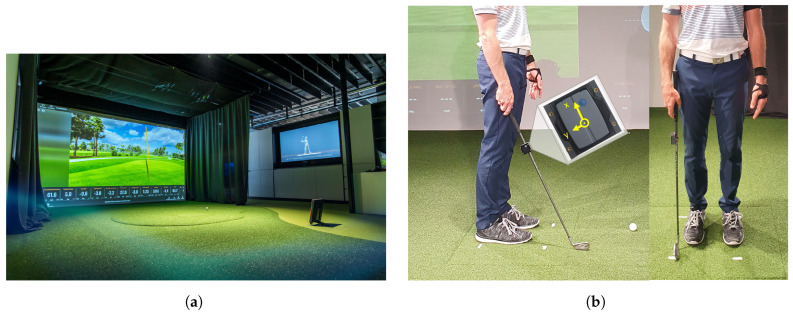
(**a**) The golf lab at MOTUM−Human Performance Institute, which hosted the experiment. The Trackman 4 is located in the optimal position right behind the tee. (**b**) The IMU sensor was mounted within a customfit 3D-printed hardshell case and positioned in axial direction 8 cm underneath the grip of the golf club.

At MOTUM−Human Performance Institute a fully equipped golf laboratory, including a Trackman 4 system, was used for the experiment (Figure 1a). During the experiment, the data from the Trackman 4 was recorded for each swing. In parallel, video data (NinoX 120, Noraxon, Scottsdale, AZ, USA, 120 Hz) and 3D-acceleration data via an IMU-Sensor (Ultium motion, Noraxon, Scottsdale, AZ, USA, 400 Hz) was recorded for each swing via the software MR3 (MyoRESEARCH 3.18.126, Noraxon, Scottsdale, AZ, USA). The IMU-Sensor was placed in a 3D-printed hardshell case and mounted 8 cm underneath the grip of the golf club (Figure 1b) in the axial direction of the club face. The Trackman 4 is capable of measuring the point of interaction of the golf club. Therefore, the data that was recorded from the Trackman was seen as the gold standard data. The IMU data, which was gathered from the Ultium motion sensors, should be used to estimate the gold standard data. A comparison of the gold standard method and the new IMU-ML approach can be derived. Hence, the setup enables the proof-of-concept for the new IMU-ML method.

**Figure 2:**
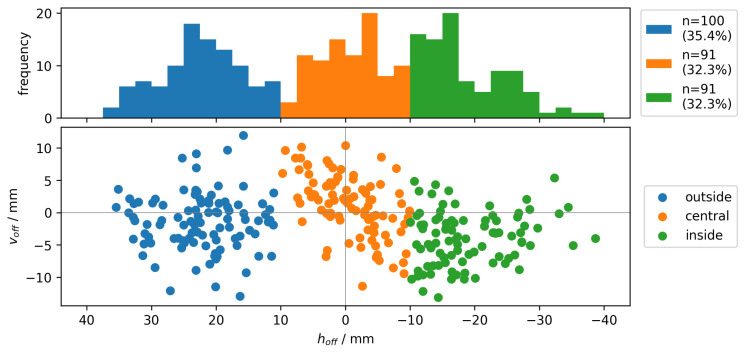
Spatial and frequency distribution of impact locations for 282 samples.

**Figure 3:**
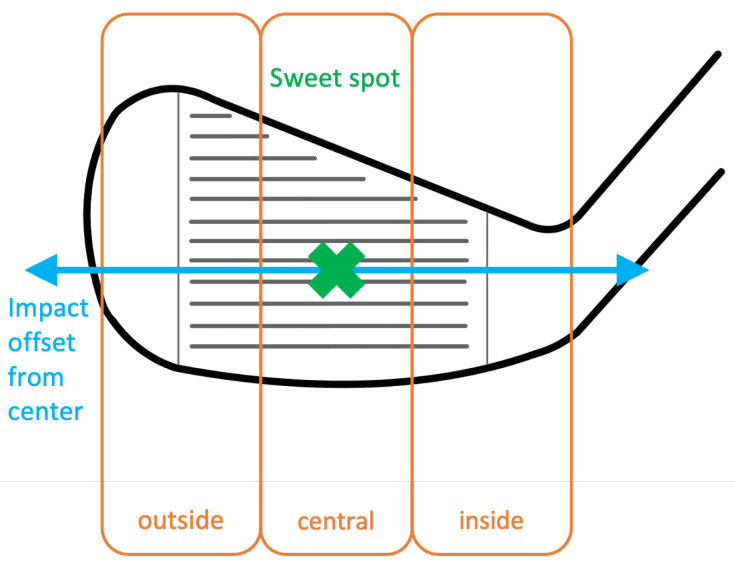
Spatial division of the impact offset from sweet spot (green cross) of the club face into three classes: central, outside and inside. The central class is defined as the area, which spans ±10 mm horizontally around the sweet spot.

As the major goal was to provide a proof-of-concept, the variability and diversity of the participants and golf gear was set to a minimum. The whole experiment was carried out with a 7 Iron (BIG MAX EMC2), one golf ball type (Titleist Ball 23 Pro V1X RCT) and one male athlete (184 cm, 78 kg). The athlete had the skill level of a professional golfer and was instructed to perform golf swings within the laboratory with the adjusted golf club. This procedure led to a wide distribution of the point of interaction between the golf club and ball, which is displayed in Figure 2. As can be seen in Figure 2 and Figure 3, three hit areas were defined with a total of 282 valid shots and an almost even distribution between classes. The impact area is spanned by a coordinate system in which the horizontal axis is defined as Impact Offset, while the center of the coordinate system is located at the sweet spot of the clubface. The three hit areas represent the final labels for the machine learning algorithm, and are divided into central, outer and inner. This division was based on Wang et al., who also used a horizontal division of the clubface in three subsections and chose a width of 17 mm for the central class [39]. The central class corresponds to an area, which spans ±10 mm horizontally ($h_{off}$) from the center of the club face. The remaining two classes represent the area around the central class, the outer class being the area further away from the club shaft and the inner class being the area closer to the club shaft. There were no further requirements set on the distribution of the hit locations.

**Figure 4:**
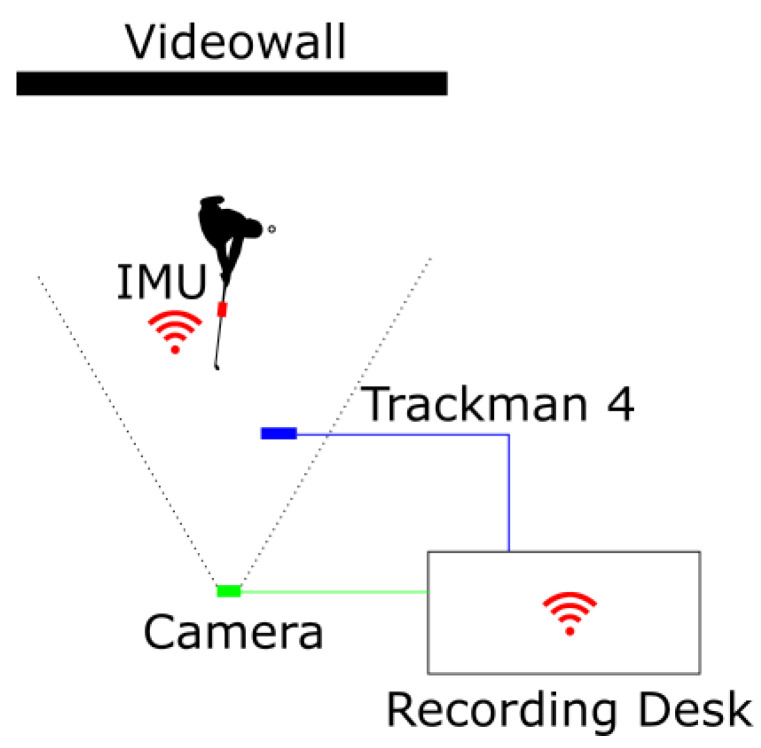
Schematic top view of the experimental setup.

While carrying out the experiment, a process for documentation and monitoring was as follows: The process ensured that the location of the Trackman 4 and the IMU on the golf club does not change over time. Each golf swing resulted in recordings of the IMU signals, data from Trackman and a video of the athlete. Figure 4 shows the experimental setup and the mounting method for the IMU on the golf club. In this setup, the IMU sensor is used with the maximum sampling rate, which is shown with all other hardware specifications in Table 1.

The data collection is divided into eleven sessions, with 20 to 30 shots in each session. Therefore, eleven .csv-files containing the IMU data were collected, each containing values for acceleration and magnetic flux densities in three axes each extended with angular velocity around these axes. This resulted in a total of nine recorded signals for each of the eleven .csv-files. One additional .csv-file was exported containing the Trackman data. For the development of an algorithm, it was necessary to further process the recorded data.

### 2.2. Data Processing

**Figure 5:**
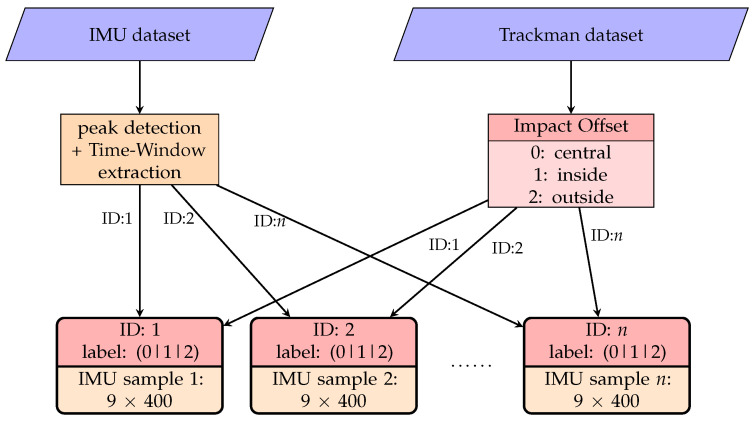
Schematic visualization of the data pre-processing.

The data pre-processing is done for each session individually using a custom Python (version 3.10.11) script utilizing the packages matplotlib, numpy, pandas, scipy and os. This step can be divided into generating time series data from the IMU data and generating a label using the Trackman data, which is displayed in Figure 5. First, each session of the IMU data were divided into shots, using a time-window of one second at 400 Hz. The time window starts 200 values before the first impact and ends 200 values after, including the value of the impact itself. Next, each shot, containing 9 × 400 values (acceleration, angular velocity and magnetic field), was normalized into values between 0 and 1 using the measurement ranges from the hardware specifications shown in Table 1. Each identified shot was labeled with an ID number to combine it with the Trackman dataset afterward. The exported .csv-file from the Trackman was used to generate the label for each shot. This was done considering the values for Impact Offset for each shot. Based on these values, each sample was classified into one of the three impact classes, which are shown in Figure 2. Label encoding is used to convert the class names into numeric labels, required for the machine learning algorithm. Therefore, central is referred to as 0, inside as 1, outside as 2. Finally, each numerical label is combined with the time-series data.

### 2.3. Data Augmentation and Splits

In order to create a suitable split of the recorded dataset for the training, validation and testing of ML architectures, 32 of 282 shots from the original dataset have been set aside and were not used during training, either as training or as validation data. This leaves a split size of 250 samples for data augmentation, which was then applied using tsaug [41] (a Python package offering various time series augmentation methods). The AddNoise and TimeWarp methods have been used to extend the number of samples from the test and validation split from 250 to 750 samples. A total of 250 samples have been created artificially, with each listed augmentation method based on the original dataset. AddNoise adds noise between 0.0 and 0.1 to each time point of the data, and is independent and identically distributed within the time series, while TimeWarp changes the speed of the timeline within one sample by changing the speeds, in this setup ten times, with a maximum speed ratio of 1.3 [41].

For the classification algorithm, the data were split in 2 folds with stratified k-Fold. The test split was created to have some data left that the optimizer has never seen. If the network is able to classify the test split correctly, it can be seen as a strong evidence for a classification that is not learn-by-heart-based. With the pre-processed and augmented dataset, the development of an ML algorithm was possible.

### 2.4. Network Architecture

As given in the data processing subsection, the input for the classification algorithm is the IMU data in the format 9 × 400. The network output should reflect the one hot encoding approach in the format of 1 × 3, with respect to the three classes. Therefore, an output was created according to the numeric label in the dataset. A numeric label ‘0’ resulted in an output vector [001], ‘1’ in [010] and ‘2’ in [001].

**Figure 6:**
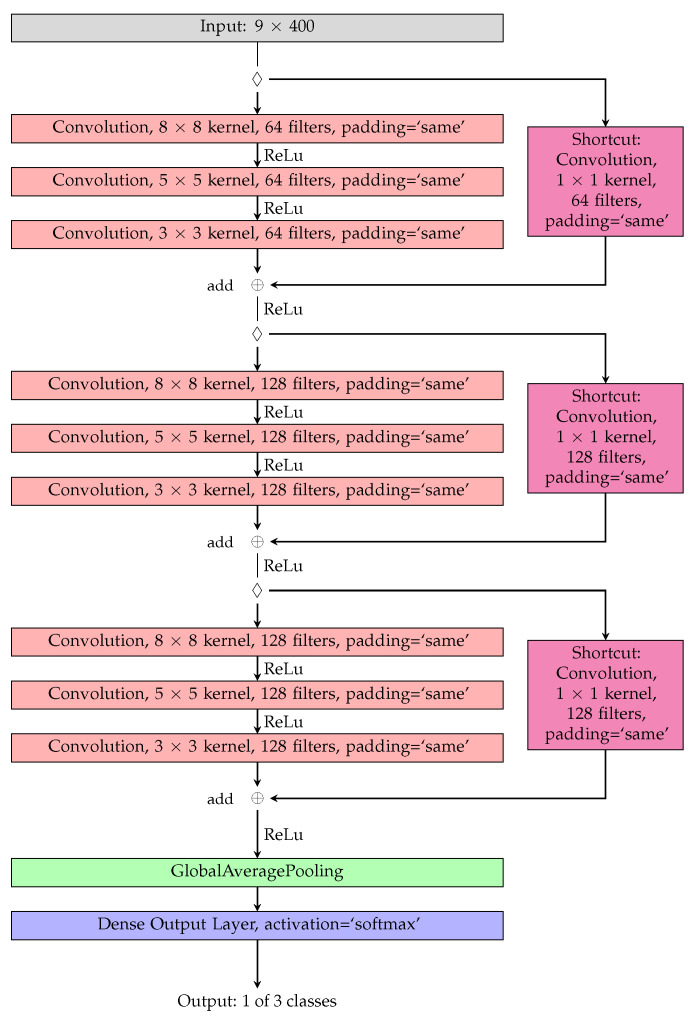
Schematic visualization of the ResNet architecture with an input size of 9 × 400 and the nine convolutional layers divided into 3 blocks for the classification into the three classes. Starting at the input feature vector, each block is connected through a shortcut connection.

For the classification algorithm, a ResNet architecture from the researchers in [23] was chosen, since it is adapted for from the original image classification competition in 2015 from [24]. The relatively deep ResNet architecture also showed promising results in [22], which has also proven its capabilities of dealing with IMU data in a sports environment, where impact and swing analytics were the goal of the classification. The chosen architecture consists of one input layer, nine convolutional layers divided into 3 × 3 convolutional blocks, and one global average pooling layer and a dense output layer with a softmax activation function. Each convolutional block has a similar structure but different number of filters. The first block has 64 filters, while the second and third block have 128 filters, each with a kernel size of eight, five and three. The first two convolutions of each block are followed by batch normalization and a ReLu activation function, while the third convolution is followed by batch normalization and the addition of the identity from the shortcut connection. The result is activated with a ReLu and forwarded into the next block. This process is continued for the next to blocks, but with different filter sizes while the shortcut connections is now connected to the previous block instead of the input layer. For a better understanding, the used architecture is provided in Figure 6.

### 2.5. Training of the Neural Network Classifiers

**Figure 7:**
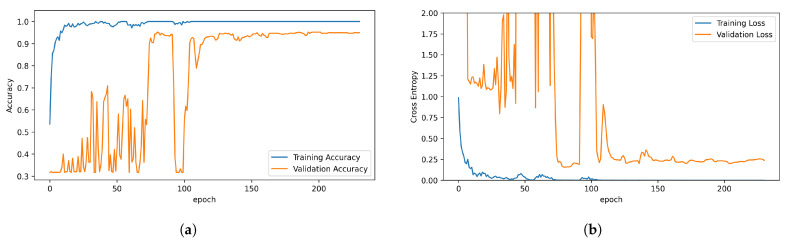
Exemplary training and validation history of (**a**) accuracy and (**b**) categorical cross entropy.

The training and evaluation of the classifiers were performed in the Google Colaboratory environment [42] utilizing a Nvidia T4 Cloud GPU (NVIDIA, Santa Clara, CA, USA), making the training of the relatively deep ResNet architecture time efficient. This resulted in a training time of 6 min and 17 s for the total of two stratified K-Folds cross validator splits [43]. This specific training and validation process is displayed in Figure 7, resulting in 230 epochs before the early stopping callback prohibits overfitting of the model. In total, the network had 510,723 trainable parameters. Adam [44] was used as optimizer with categorical cross entropy as loss function.

## 3. Results

**Figure 8:**
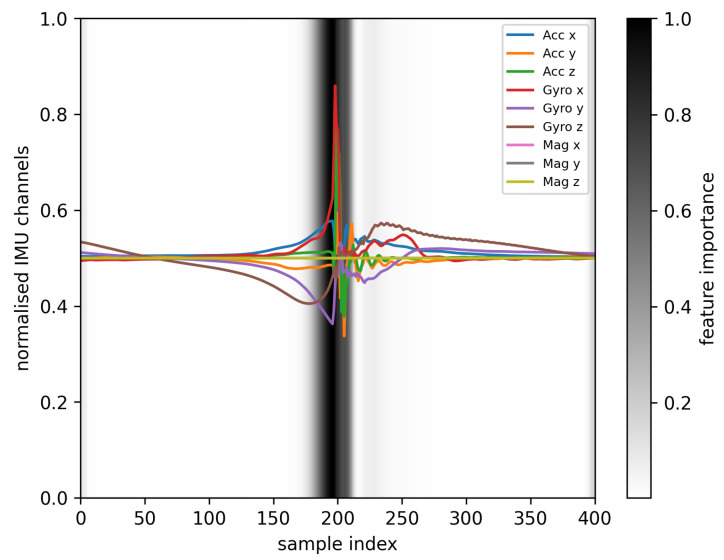
Feature importance heatmap for one IMU sample, where the time-dependent importance of the IMU sample to the trained ML network is displayed as a grayscale heatmap. Values of zero (white) correspond to a low importance, while the value one (black) represents high importance.

**Figure 9:**
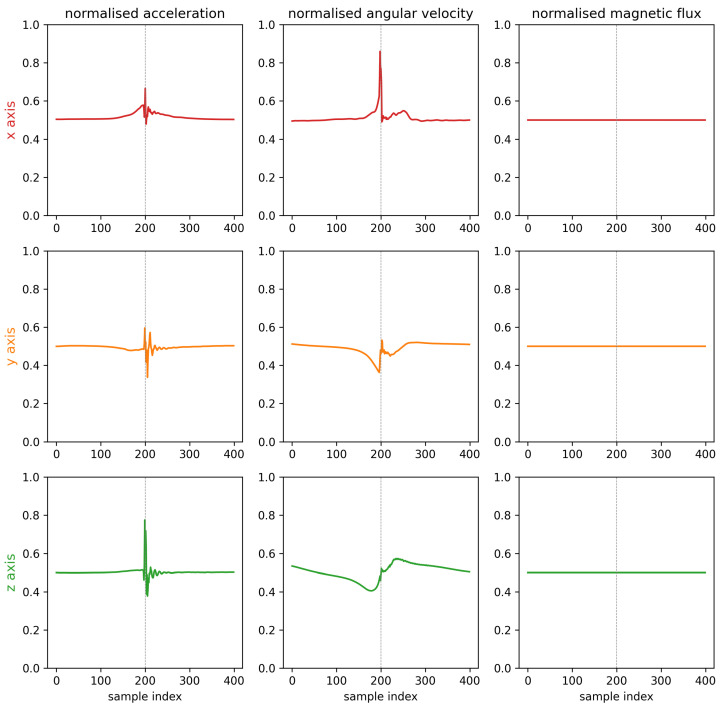
IMU data sample of a performed impact, with the impact timing around the sample index 200, highlighted as a dotted line. The measured values are normalized to values between 0 and 1 using the hardware configuration (Table 1).

First, the results of the IMU measurements and the performance of the impact offset classification on the test dataset is provided. Further results show the connection between the trained network, its weights or detected features and the IMU data. Therefore, a feature importance heatmap is provided in Figure 8, which displays the same IMU sample as in Figure 9.

In Figure 9, a sample of the IMU data are provided. It displays all nine degrees of freedom (DoF) of the IMU sensor, normalized in the range between 0 and 1 using the maximum measurement ranges supplied in Table 1. The normalized acceleration for the x- and y-component show slight deviations around 0.5 (default state) before the impact, which occurs around the sample index 200. At the impact, all three dimensions shows a peak followed by a decaying oscillation. The peak is maximal at the z-component due to the orientation of the z-axis of the IMU sensor in impact or ball flight direction, as shown in Figure 1b. The normalized angular velocity shows a peak around the sample index of the impact, especially in the x-component. The z-component is smoother than the x- and y-component and forms, besides the noise due to the impact, a shape that is point-mirrored at the sample index of the impact. The normalized magnetic flux in x-, y- and z-component do not show any visual changes in the sample, when normalized to the measurement ranges of the IMU sensor. This is caused by the ratio of the near-ground geomagnetic field at the measurement location (which ranges between 0.25 and 0.65 Gauss on earths surface [45]) to the sensor maximal measurement ranges displayed in Table 1.

**Figure 10:**
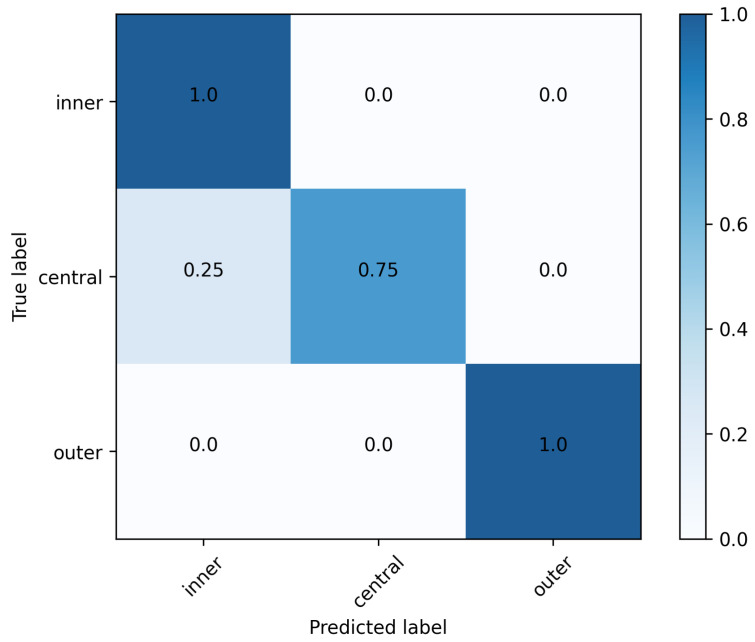
Normalized confusion matrix for performance evaluation of the classification network.

The results for the impact offset classification for the test data are displayed in Figure 10 using a confusion matrix. The network, which is shown in Figure 6, provides a maximal accuracy of 93.8% on the test set containing 32 unseen IMU samples.

To bring the results of the displayed sample in Figure 9 into context with the performance of the classification network, Figure 8 shows the feature importance as an overlay on the nine DoF IMU sample. The heatmap is color graded, ranging from 0 (white) to 1 (black). A value of one in feature importance shows the time-dependent importance of the IMU data to the trained ML network. In Figure 8, a high feature importance is detected when the first peaks in the angular velocity of the gyroscope are detected. The intensity is evenly distributed around the highest values for the gyroscope measurement (x-component) and decreases to both sides of the maximum. All other areas for samples indices 0 to 175 and 225 to 400 do not show a visually detectable feature importance.

## 4. Discussion

The study presents a novel approach to classify the impact location between a golf club and ball using an IMU sensor mounted to the golf club’s shaft.

When comparing this study to prior research and other devices [38], the approach of using a IMU sensor in a mobile setting to receive information on the impact offset is new to golf. Therefore, the aim was to conduct a feasibility study to create a proof-of-concept. The results show maximal accuracy of the classification network of 93.8%, indicating a general success of the proposed methods and therefore also the feasibility. This approach forms a high agreement of the predictions of the IMU-ML model with the measured gold standard Trackman 4 data. With this approach, a framework is set to further investigate the usage of IMU system as direct mobile feedback system attached to the golf club and to address the disadvantages of stationary methods.

While the Trackman 4 is used as a reference for class labeling, it is important to consider the possibility of slight deviations from the actual point of impact. As the impact location (inside of the sweetspot, central around the sweetspot and outside of the sweetspot) will influence the vibration characteristics, these three labels were chosen. There may be cases where the measured values closely match the boundary between two classes (see Figure 3), which underlines the importance of refining the class boundaries to reduce possible misclassifications. Potentially, regression models can allow the identification of the impact distance from the sweetspot, instead of the very general area identification described in the current paper. However, this was not scope of the current paper, which aimed to focus on the feasibility of class identification.

Additionally, we found that a 400 Hz sensor was in the current dataset sufficient to detect the impact location within the three predefined locations with the given accuracy. This supports our assumption that the impact location mainly affects the vibration characteristics, rather than the impact peak.

The relatively small size of the dataset, while suitable for validating proof of concept, raises concerns about generalizability. The consistency of the golfers’ strokes, combined with the exclusive use of a specific club and ball combination, contributes to the high degree of specificity of the data set. To increase external validity, future research should aim to include a wider range of golfers, swing styles and equipment configurations. The use of a stratified K-fold approach to split the test set ensures a representative distribution of samples across the different impact offset classes. However, the limited size of the test set leads to an inherent class imbalance, particularly in the outer class. This imbalance should be taken into account when interpreting the performance metrics of the model.

An ablation study, displayed in Table 2, was conducted to assess the contribution of each sensor to the accuracy of the test dataset. The data of the ablation study was obtained reducing the amount of all relevant DoF of the input data, resulting in four configurations. The first configuration provides all nine DoF while the configuration two, three, and four only show the network performance for the accelerometer, gyroscope, and magnetometer, respectively. The median accuracy, calculated over five iterations, is used as a performance metric. For configurations 2–4, the unused DoFs were set to zero, resulting in no changes to the input size and the number of trainable parameter for the network. This study provides valuable insights. In particular, the magnetometer proved to be inconclusive as to the location of the impact. In contrast, both the accelerometer and the gyroscope provided significant contributions, highlighting their central role in the classification process. This result suggests that the measurement methodology could be simplified by using an IMU sensor with only the acceleration and gyroscope signals. As a consequence, it would lead to a reduction in the input dimensions for the ResNet network architecture, allowing the model to have less trainable parameter with similar performance. All sensor inputs that are marked with a cross in Table 2 are used for this iteration.

The results of the ablation study are supported by the direct observation of the magnetometer’s normalized values in Figure 8. There are no visible changes in the measured values, which underlines the low relevance of the magnetometer for the accuracy of the network. Additionally, the feature importance heatmap provides visual representation of the time-dependent importance of the IMU data to the trained ML network. This representation allows for analyzing of the relevant time-window for the ML network. The results indicate that the most significant feature can be found between sample indices 175 and 225 (0.125 s), which is around the sample index of the impact. This would allow future research to focus on this specific time-window, only using the measured values from the accelerometer and the gyroscope, to reduce input sizes for the ML network.

## 5. Conclusions

In summary, this study presents an innovative approach to accurately classify the point of impact between the golf club and the ball using an IMU sensor attached to the shaft of the golf club. The results show a noteworthy success rate, with the classification network achieving an accuracy of up to 93.8%. Further examination of the IMU data, coupled with an exploration of the temporal significance of the features, indicates that the acceleration and gyroscope data are of notable significance, while the magnetometer data are of comparatively minor relevance. Consequently, it is suggested that subsequent investigations consider a refined temporal resolution, possibly reducing the analyzed time window from one second to 0.125 s. This highlights the effectiveness of the proposed methodology, and confirms the feasibility of using IMU sensors in a mobile environment for golf shot analysis. Moving on, more golf-specific performance parameters, such as the vertical component of the impact location, attack angle, face angle, swing direction and many more, describing either the movement of the club, the interaction between club and ball, or the resulting movement of the ball after the impact, can be investigated using the IMU-ML approach. The IMU-ML approach could improve the ability to capture critical biomechanical parameters during golf swings, but not limited to a laboratory setting and the use of expensive and inaccessible equipment. This creates the opportunity of a mobile and personalized measurement system that can provide the user with extensive information on golf-specific performance metrics. Such a system allows continuous monitoring of progress, enabling golfers to improve their skills through data-driven approaches.

Suggestions for a future steps involve a fine-tuning of the existing ResNet algorithm, further and broader data acquisition and a consideration of using an ML regression model, to not only predict the impact offset classes but predict the actual impact location as an offset, enabling an even deeper information transfer to the athlete. By deriving the results of the ablation study, the degrees of freedom of the measurement system could also be reduced. It is now necessary to further simplify the measurement setup, e.g., by recording with other measurement frequencies, and by using other IMU units, which not only have other maximum values of the respective sensors, but also other sizes and weights. If the idea of a mobile golf analysis tool is pursued further, the attachment of the sensor to the shaft must also be revised. One solution for this would be to install it directly in the club shaft, accessible at the top of the grip.

## Figures and Tables

**Table 1:** Hardware specifications of Noraxon Ultium IMU Sensor [40].

Measurement	Measurement Ranges	Maximum Measurement Output in Hz
Acceleration	±200 g	400
Angular velocity	±7000 deg/s	400
Magnetic field	±16 Gauss	100

**Table 2:** Ablation study with reduction in DoF. The median accuracy over five iterations is displayed. All uncrossed signals were zeroed for the specific configuration to have no change to the network’s input size and its number of trainable parameters.

Configuration	Accelerometer	Gyroscope	Magnetometer	Median Accuracy
1	✗	✗	✗	87.5%
2	✗			84.4%
3		✗		81.2%
4			✗	28.1%

## Data Availability

The data presented in this study are available on request from the corresponding author.

## Abbreviations

The following abbreviations are used in this manuscript:

MCI	Management Center Innsbruck
IMU	inertial measurement unit
ICA	independent component analysis
etc.	et cetera
ADC	analog digital converter
e.g.,	exempli gratia
CNN	convolutional neural network
LSTM	long short time memory
ReLu	rectified linear unit
ML	machine learning
FC	fully connected
DoF	degree of freedom
